# Evaluation of an Intrahospital Telemedicine Program for Patients Admitted With COVID-19: Mixed Methods Study

**DOI:** 10.2196/25987

**Published:** 2021-04-29

**Authors:** Sean Legler, Matthew Diehl, Brian Hilliard, Andrew Olson, Rebecca Markowitz, Christopher Tignanelli, Genevieve B Melton, Alain Broccard, Jonathan Kirsch, Michael Usher

**Affiliations:** 1 Division of General Internal Medicine Department of Medicine University of Minnesota Medical School Minneapolis, MN United States; 2 Department of Surgery University of Minnesota Medical School Minneapolis, MN United States

**Keywords:** telemedicine, hospital medicine, COVID-19, telehealth, hospital, mixed methods, evaluation, impact, exposure, risk, communication

## Abstract

**Background:**

The increasing incidence of COVID-19 infection has challenged health care systems to increase capacity while conserving personal protective equipment (PPE) supplies and minimizing nosocomial spread. Telemedicine shows promise to address these challenges but lacks comprehensive evaluation in the inpatient environment.

**Objective:**

The aim of this study is to evaluate an intrahospital telemedicine program (virtual care), along with its impact on exposure risk and communication.

**Methods:**

We conducted a natural experiment of virtual care on patients admitted for COVID-19. The primary exposure variable was documented use of virtual care. Patient characteristics, PPE use rates, and their association with virtual care use were assessed. In parallel, we conducted surveys with patients and clinicians to capture satisfaction with virtual care along the domains of communication, medical treatment, and exposure risk.

**Results:**

Of 137 total patients in our primary analysis, 43 patients used virtual care. In total, there were 82 inpatient days of use and 401 inpatient days without use. Hospital utilization and illness severity were similar in patients who opted in versus opted out. Virtual care was associated with a significant reduction in PPE use and physical exam rate. Surveys of 41 patients and clinicians showed high rates of recommendation for further use, and subjective improvements in communication. However, providers and patients expressed limitations in usability, medical assessment, and empathetic communication.

**Conclusions:**

In this pilot natural experiment, only a subset of patients used inpatient virtual care. When used, virtual care was associated with reductions in PPE use, reductions in exposure risk, and patient and provider satisfaction.

## Introduction

The COVID-19 pandemic presents unprecedented challenges to infection control in hospitals worldwide. These challenges are particularly urgent given the potential exposure risk for both health care personnel and uninfected patients amid shortages of personal protective equipment (PPE).

The health care–associated spread of COVID-19 to patients and health care workers is well documented. In Wuhan, China, presumed hospital-related transmission of COVID-19 was suspected in 41% of patients [1]. In Italy, 20% of health care professionals in one health system became infected with COVID-19 [2]. In Washington state, lack of sufficient PPE use in a skilled nursing facility is thought to have contributed to the spread of infection to 101 residents, 34 staff members, and 14 visitors [3]. As of September 2, 2020, nearly 570,000 health care providers in the Pan-American region have been infected with COVID-19 and more than 2500 have died [4]. Although health care providers may be infected outside their job duties, health care–associated COVID-19 infections are potentially avoidable, cause direct morbidity and mortality, and lead to a reduction in workforce capacity as many health care workers are quarantined. Addressing these risks, the World Health Organization and Centers for Disease Control and Prevention both recognize PPE as one of the most effective preventive measures to reduce transmission of COVID-19. Unfortunately, there is currently a global shortage due to a surge in demand and disruptions to the global supply chain [5,6]. Although strategies have been developed to mitigate these shortages and infection risks, they will continue to present challenges as the pandemic continues.

Telemedicine has been proposed as a potential innovative solution to reduce the infection risk associated with COVID-19 [7-9]. Telemedicine, including delivery in a hospital-based setting, has the capacity to facilitate communication and a visual examination of a patient without entering the room. This in turn has the potential to decrease PPE use and health care–associated COVID-19 transmission. More than 50 US health systems already have existing telemedicine programs [7], which have been used in ambulatory [10], triage [11], inpatient [12], and intensive care unit settings [13]. These programs are associated with high patient and provider satisfaction and noninferior clinical outcomes [12,14,15]. Furthermore, telemedicine has been shown to be feasible in the context of the COVID-19 pandemic [16-19]. To date, however, telemedicine in the hospital-based setting has typically centered on using this technology to increase coverage to critical access hospitals—often for specialist services not available locally (eg, intensivists)—or for consultants as opposed to a more ubiquitous care modality for inpatient care.

To our knowledge, telemedicine in the hospital has not been extensively used and studied with the explicit purpose of reducing infection exposure and PPE use. If intrahospital telemedicine (virtual care) is an option, providers and patients have a choice on whether to conduct an in-person evaluation or virtual evaluation for a given interaction. This creates a unique environment to test the impact of a novel use for telemedicine technology on communication, in addition to evaluating its impact on exposure risk and PPE use and patient experience.

We built an in-house virtual care system at a large academic medical center in the midwestern United States with a dedicated COVID-19 service. The primary objective of this study was to evaluate if virtual care for hospitalized patients with confirmed COVID-19 could reduce overall exposures to patients with COVID-19. Secondary outcomes included patient and provider satisfaction.

## Methods

The study was a prospective cohort study conducted in three general medicine units at an urban university hospital solely dedicated to treating patients with a confirmed positive test for COVID-19. The chart review portion of the study was approved by the University of Minnesota Institutional Review Board.

### Patients

All patients were confirmed to have a positive polymerase chain reaction test result indicating infection with COVID-19 prior to transfer to the COVID-19–specific hospital to one of three medicine units. Only adult patients over 18 years of age were included in the evaluation.

### Intervention

We conducted a natural experiment of an intrahospital telemedicine program (termed virtual care) on three medical units in a dedicated COVID-19 hospital. All rooms were equipped with an iPad (Apple Inc) with third-party videoconferencing software (Polycom) assigned to the patient’s room. Patients and providers were given the option to opt out of the use of this virtual technology on a daily basis if deemed inappropriate based on mental status, illness severity, technical issues, or individual preference. If accepted for use, providers and nurses could conference into the patient’s room using a device at the nursing station or an enterprise-associated phone or tablet. The virtual evaluation process required the patient to opt in by either leaving a virtual room open or turning on the camera and microphone. We present an analysis of a convenience sample of consecutively collected patients during a 6-week evaluation period.

### Measures

Age, race, gender, interpreter use, and comorbidities were extracted from the electronic health record (EHR). Comorbidities were characterized by the Elixhauser Comorbidity Index using diagnosis codes collected in the year prior to hospitalization [20]. In addition, iPad use was reported in a standardized EHR note template, which was tracked on a daily basis prospectively. We measured PPE use on even number days by extracting daily PPE usage logs that were placed on each patient’s door and filled out by all staff entering the room. Admission days, discharge days, and days where the patient was transferred between units were excluded. We specifically report face shield, mask, and gown use rates. We also report PPE breach rates, which were recorded on the same sheet. Finally, we report physical exam rates, which is the number of in-person exams conducted by physicians and nurses documented in the chart or sign-in sheet.

### Survey Deployment

We developed a multidisciplinary workgroup to assess patient, nurse, and physician satisfaction. The goals were to assess comfort with the technology, usefulness in supporting communication, disruptiveness, and perceptions of safety using a modified Likert scale. Questions were designed to adhere loosely to the Technology Acceptance Model [21], assessing perceived usefulness, perceived ease of use, and attitude toward use. Clinicians serving dedicated COVID-19 units were contacted with both paper and electronic surveys. Patients were contacted virtually via the iPad or by phone if they had at least one documented use of virtual care and were felt to be close to discharge by the patient’s medical team. Surveys were continually applied to patients as they approached discharge, and were distributed to providers and nurses at week 3 and week 6. At week 6, an additional survey was distributed to providers and nurses to address usability and capture reasons inpatient virtual care was not used.

### Statistical Analysis

Continuous variables are displayed as median and IQR for skewed variables or otherwise mean and SD and binary or categorical variables as a count and percentage. An ordinal variable was generated related to survey responses as measured by 5-point Likert scale, with 0 being strongly disagree and 4 being strongly agree. Mean and SD for each group (patients, clinicians) were calculated. Between-group comparisons between patients who opted in and opted out were performed using Mann-Whitney and chi-square tests where appropriate.

To adjust for repeated measures, daily PPE use rates were converted into panel data. Gown, face shield, and mask use were evaluated using Poisson regression adjusting for clustering by patient. Physical exam rate was evaluated by negative binomial regression. Two comparisons were performed. First, we evaluated the impact of virtual care by evaluating per-patient daily PPE use against all control patient days. Since patients were not reported to use virtual care every day, we performed a subsequent sensitivity analysis comparing PPE use on days where virtual care was used against days it was not, among a cohort that used it at least once. All statistical analysis was completed in STATA (version 16; StataCorp LLC).

## Results

### Overview

A convenience sample of 137 patients admitted to the COVID-19 inpatient unit was evaluated, accounting for a total of 483 patient days during the 6-week evaluation period. This included 43 patients who used virtual care at least once (Table 1). On average, patients who opt out versus opt in to virtual care are not statistically different with respect to age (mean 66.6 years versus 65.1 years, *P*=.62), gender (46% male versus 49% male, *P*=.79), racial make-up (62% White versus 49% White, *P*=.15), or comorbidity rates. Although proportionally more White patients opted out of virtual care than Black or Asian patients, there was not a statistically significant difference. Use of interpreters was lower in the opt-in population (12% versus 20%, *P=*.09), but this did not reach statistical significance. On average, those patients who opted in had a longer length of stay (11.8 days versus 9.9 days, *P=*.09) but this was also not statistically significant. Similarly, there were no significant differences in mortality in those who opted in versus opted out (7% versus 10%, *P=*.62) or maximum oxygen requirements (maximum oxygen requirement 4.5 liters per minute versus 6 liters per minute, *P=*.68).

**Table 1:** Patient demographics, comorbidities, and hospital utilization among patients with and without virtual care.

Variables		Opt-out group (n=94)	Opt-in group (n=43)	*P* value
**Demographics**				
	Age years, mean (SD)	66.6 (18)	65.1 (19)	.62
	Male, n (%)	46 (49)	20 (46)	.79
	White, n (%)	58 (62)	21 (49)	.16
	Black, n (%)	14 (15)	8 (19)	.58
	Asian, n (%)	10 (11)	4 (9)	.81
	Other/missing, n (%)	12 (13)	10 (23)	.12
**Comorbidities**				
	Congestive heart failure, n (%)	26 (28)	7 (16)	.08
	Chronic obstructive pulmonary disease, n (%)	15 (16)	5 (12)	.50
	Obesity, n (%)	26 (28)	16.9 (37)	.26
	Depression, n (%)	27 (29)	16 (37)	.32
	Hypertension, n (%)	68 (72)	30 (70)	.76
	Diabetes mellitus, n (%)	29 (31)	17 (40)	.32
	Elixhauser Comorbidity Index score, median (IQR)	5 (3-8)	5 (3-7)	.49
**Hospital utilization**				
	Maximum oxygen requirement (liters per minute), median (IQR)	6 (3-10)	4.5 (3-7)	.68
	Discharged, n (%)	70 (74)	31 (72)	.77
	Length of stay (days, if discharged), median (IQR)	9.9 (6.5-14.9)	11.8 (7.0-24.9)	.09
	In-hospital mortality, n (%)	9 (10)	3 (7)	.62
	Interpreter use, n (%)	19 (20)	5 (12)	.09

### Use of PPE

Distribution of daily PPE use is displayed in Figure S1 in Multimedia Appendix 1. As shown in Table 2, daily gown use (median 5, IQR 4-5 versus median 3, IQR 2-3; *P*<.001), face shield use (median 5, IQR 4-5 versus median 3, IQR 2-3; *P*<.001, *P*=.001), and mask use (median 5, IQR 4-5 versus median 3, IQR 2-3; *P*<.001) were all reduced on days where virtual care was reported to be used. Similarly, physical exam rates (median 1, IQR 1-2 versus median 0, IQR 0-1; *P*<.001) were reduced on days where virtual care was used. There was no significant difference in the recorded PPE breach rate; breaches were rare for both groups. Similar differences were found when comparing days where virtual care was used and not used among patients who opted in (Table 2).

**Table 2:** Daily personal protective equipment use rates among patients with and without virtual care.

Variables		Opt-out group	Opt-in group	Coefficient (95% CI)	*P* value
**All patients**					
	Number of days	401	82	N/A^a^	N/A
	Face shields, median (IQR)	5 (4-6)	3 (2-3)	–0.54 (–0.65 to –0.43)^b^	<.001
	Masks, median (IQR)	5 (4-6)	3 (2-3)	–0.54 (–0.65 to –0.43)^b^	<.001
	Gowns, median (IQR)	5 (5-6)	3 (2-3)	–0.54 (-0.65 to –0.43)^b^	<.001
	Physical exam, median (IQR)	1 (1-2)	0 (0-1)	–0.84 (–1.12 to –0.57)^c^	.001
	Personal protective equipment breach, n (%)	4 (1)	3 (4)	1.65 (0.33 to 8.30)^d^	.55
**Patients who opted in at least once**					
	Number of days	116	82	N/A	N/A
	Face shields, median (IQR)	5 (4-6)	3 (2-3)	–0.54 (–0.69 to –0.41)^b^	<.001
	Masks, median (IQR)	5 (4-6)	3 (2-3)	–0.54 (–0.69 to –0.41)^b^	<.001
	Gowns, median (IQR)	5 (5-6)	3 (2-3)	–0.54 (–0.69 to –0.41)^b^	<.001
	Physical exam, median (IQR)	1 (1-2)	0 (0-1)	–0.85 (–1.31 to –0.56)^c^	.001
	Personal protective equipment breach, n (%)	2 (2)	5 (6)	1.65 (0.33 to 8.30)^d^	.55

^a^N/A: not applicable.

^b^Poisson regression adjusting for clustering by patient.

^c^Negative binomial regression adjusting for clustering by patient.

^d^Logistic regression adjusting for clustering by patient.

### Patient Experience

We then investigated patient attitudes and perceptions on use and usability. The overall response rate was 40% (42/105), and 65% (27/41) for providers. Among patients surveyed, 8 of 15 (53%) reported common use of videoconferencing and 5 (33%) reported not using it at all. Overall, the patients’ perceptions of virtual care were positive (Table 3). Furthermore, 8 (53%) respondents reported virtual care improved communication with the care team; however, only 7 (46%) felt emotionally supported through virtual communication. Patients reported improvements in sense of isolation with use (11/15, 73%), felt that it reduced exposure risk (n=15, 100%), and felt that overall continuing the project was a good idea (n=14, 92%). Importantly, patients largely felt that providers remained responsive to patient needs (n=14, 92%), and 10 (67%) disagreed with the idea that virtual care would cause doctors to miss something.

**Table 3:** Patient satisfaction: means and distributions of survey responses of patients with COVID-19.

Survey questions	Mean (SD)^a^
I use Facetime or other video chat programs in my daily life	2.13 (1.50)
The purpose and use of the iPad was explained to me	2.53 (1.12)
My doctor and nurses primarily communicated with me virtually	1.73 (1.10)
Virtual visits improved my ability to communicate with my care team	2.73 (0.96)
I felt that use of virtual communication reduced the risk of exposing my medical team to COVID-19	3.60 (0.51)
My care team was able to emotionally support me through virtual communication	2.27 (0.96)
Being able to use virtual communication allowed me to feel less isolated	2.93 (0.80)
My care team was responsive to my needs including visiting me in person when I needed it	3.67 (0.62)
I feel that my care team was more likely to miss something when relying on virtual communication	1.33 (0.82)
I think continuing to use virtual visits in the hospital is a good idea	3.13 (0.52)

^a^Modified Likert scale was converted to a 0-4 scale, with 0 denoting strongly disagree and 4 denoting strongly agree. Scores above 2 are significantly more positive.

### Health Care Worker Experience

Results of the provider survey are shown in Table 4. Two areas identified as potential barriers for provider acceptance of this technology were lack of prior use of telemedicine with patients (Likert mean 1.65, SD 1.44) and concern that virtual care does not allow for adequate assessment of patient disease severity (Likert mean 1.92, SD 1.02; responses less than 2 indicated disagreement). Benefits of virtual care included reduced exposure risk and PPE use for providers (Likert mean 3.19, SD 0.85) and interest in expansion of virtual care visits (Likert mean 3.09, SD 0.7). Providers on average were less confident in virtual care’s ability to allow effective evaluation of disease severity (n=7, 26% responding positively) and empathetic communication (n=11, 41% responding positively).

**Table 4:** Provider satisfaction: means and distribution of surveys to health care workers caring for patients with COVID-19.

Survey questions	Mean (SD)^a^
I am familiar with telemedicine technology	2.69 (0.93)
I had personally used telemedicine with a patient prior to this initiative	1.65 (1.44)
Using the iPad/remote assessment improves my job effectiveness and performance	2.38 (1.06)
Using the iPad/remote assessment makes me feel safer	2.65 (0.98)
Using the iPad/remote assessment reduced my exposure risk and use of personal protective equipment	3.19 (0.85)
Using the iPad/remote assessment is disruptive to my normal workflow	1.84 (1.00)
Using the iPad/remote assessment improves my ability to communicate with patients under isolation	2.69 (0.97)
Using the iPad/remote assessment allows for adequate assessment of patient disease severity	1.92 (1.02)
Using the iPad/remote assessment allowed me to effectively show empathy to patients under isolation	2.15 (1.12)
iPad/Remote assessment should be expanded to other patients with COVID-19	2.84 (0.96)

^a^Modified Likert scale was converted to a 0-4 scale, with 0 denoting strongly disagree and 4 denoting strongly agree.

Finally, we investigated barriers among providers who were not successful in using virtual care (Table S1 in Multimedia Appendix 1). Sentiment for virtual health remained positive in regard to improving communication, family involvement, and reducing exposure risk (Table S2 in Multimedia Appendix 1) even among providers who reported difficulty with use. Respondents reported language (n=7, 58% reported frequent occurrence) and usability issues (n=8, 67% reported frequent occurrence) were the most common barriers to use. The virtual care also did not appear to detract significantly from providers’ efficiency; most disagreed with the statement that the iPad/remote assessment was disruptive to normal workflow (Likert mean 1.84, SD 1.0). Patient and provider preference as a barrier to use was also rare (Table S1 in Multimedia Appendix 1).

## Discussion

### Principal Findings

In the era of COVID-19, hospitals have necessarily become testing grounds for innovation. Inpatient wards under duress need to adapt to patient surges, minimize health care worker exposure, prevent nosocomial transmission, and streamline patient flow. Despite these pressures, however, the human core of medical care—effective communication, empathy, and clinical expertise—should not be sacrificed. We took a mixed methods approach to evaluate the degree to which an inpatient telemedicine program (virtual care) could reduce COVID-19 exposure risk and PPE use while maintaining effective communication. Although telehealth programs have rapidly expanded in the context of COVID-19, direct evaluation of patient and provider experience and overall utility remain understudied. This is particularly true for inpatients where choice between a virtual encounter and in-person encounter exists continuously. We provide several contributions to the literature that have important implications.

We provide a structured evaluation of virtual care for patients with COVID-19 and find important limitations. Patients and providers had the ability to opt out of use based on usability, preference, and medical appropriateness. As such, it was used a minority of the time even when it was available for all patients. Even among patients who used virtual care, it was relied upon less than half the days. Providers found use was of limited utility for risk assessment and providing empathetic communication.

Concerns regarding empathetic communication echo the findings that the digitization of health care can lead to a corresponding decrease in the expression of empathy by providers [22]. For example, in at least one study, empathy and praise utterances were observed less in telemedicine consultations compared to face-to-face consultations [23]. This is of particular importance because empathy is a fundamental determinant of quality in medical care [24]. Techniques have been suggested to overcome these barriers during the COVID-19 pandemic, such as finding a private place for videoconferencing, looking directly at the camera, ensuring proper lighting, and paying close attention to subtle comments and body language [16,25]. However, it is not yet clear whether these or other measures can fully bridge the digital empathy gap.

These observations highlight the importance of patient and provider engagement in the implementation of new technology, especially during a crisis. In this natural study, implementation was guided by efficiency to address a rapidly emerging health crisis. Development of a more patient-centered telemedicine program could build upon this work, improving accessibility by integrating translation services, addressing the needs of those who are hard of hearing or have vision loss, and integrating other health services to promote multidisciplinary virtual rounding. Without a patient-centered approach, there is also a risk that the digital health divide could exacerbate health disparities even within the confines of the hospital [26]. It is estimated that in the United States, not only may 13 million older adults have trouble accessing telemedical services during the COVID-19 pandemic, but also a disproportionate number of those may be among the already disadvantaged [27]. This study thus provides a first step toward understanding what is needed for a more patient-centered system.

Despite these limitations, providers and patients were generally positive regarding their experience with inpatient virtual care in two domains: supporting patient-provider communication and reducing risk of exposure. Both patients and providers supported the value of a virtual rounding program for COVID-19, with all patients and 73% (19/26) providers surveyed agreeing with program continuance. This is consistent with prior literature, which has found adequate patient satisfaction with telemedicine interventions, including during the COVID-19 pandemic [9,11,15,16,25,28,29].

Importantly, virtual care was also associated with significantly reduced PPE usage and potential exposure. Given critical shortfalls in PPE supply and distribution, efficient management of “burn rate” is an important factor in maintaining safety in our health care system [5,6]. This observation was corroborated by survey data in which both providers and patients agree that virtual care reduced exposure and PPE use. Understanding predictors of use and identifying factors that can improve efficiency of PPE use will be an important future area of study in adapting to the COVID-19 pandemic [30]. It is not known, however, whether the reduced exposure alone led to a subsequent decrease in health care–associated COVID-19 transmission because health care–associated infections were not reported as part of this study.

There are several further limitations to this study as a single-center natural experiment on the use of inpatient telemedicine. First, the sample size is small and as such we may not be able to capture the full variation in PPE use pattern or patient experience. Future work with larger sample sizes should elucidate in which ways patient satisfaction varies based on demographic groups. Second, this was not a randomized trial and there are numerous possible sources for bias. Associations can be confounded by unobserved factors such as policy changes in PPE use (such as approaches to reuse) and patient characteristics. One alternative explanation for our findings, for example, is that the differences in PPE use could be related to changes in illness severity during times in which inpatient telemedicine was felt to not be medically appropriate. Although a survey of clinicians who reported usability barriers suggested this was a minority of episodes, such a relationship could still be present. A critical next step will be understanding how PPE use rates correlate with patient factors and how they change temporally, while also determining how they correlate with outcomes.

Similarly, as this experiment used new technology, lack of use may be highly practitioner dependent and less dependent on patient-related factors. Thus, observed PPE differences and patient experiences could be attributed to the providers that tended to use virtual care more often. More structured evaluation of use to eliminate some of these inherent biases with our observational experience, such as a cluster randomized controlled trial with structured use protocols, would be better designed to overcome these shortfalls. Finally, we only surveyed patients who used virtual care at least once. The primary reason for this design was to get perspectives based on direct experience. As such, we did not obtain perceptions for patients who did not choose to use virtual care, resulting in an additional source of potential bias, and limitation to generalizability of our study findings.

Importantly, this program was built upon pre-existing technology available to our health care system, including iPads, an enterprise network, and Health Insurance Portability and Accountability Act–compliant videoconferencing technology. To other health care systems without existing telemedicine infrastructure, this intervention may be more expensive and difficult to put into place. Furthermore, in the setting of this study, a direct in-person evaluation was always immediately available. In such circumstances, intrahospital telemedicine may not be directly comparable to a fully remote telemedicine model where in-person consultation is not possible.

Indeed, the psychological effects of virtual care possibilities in light of COVID-19 warrant further investigation. We found gaps in the ability to deliver empathetic care, but there were also anecdotal stories of success. For example, an additional use case that was used was the ability to hold remote family conversations for patients in isolation. Additionally, we had multiple family members hospitalized in separate rooms in this study who could stay continually connected through this technology, for which they expressed gratitude during our survey collection.

Ultimately, we suggest that an individualized, prudent selection process for the use of virtual care may offer an appropriate use model in response to COVID-19. This can be based on evolving and unique patient and provider characteristics. In times of clinical stability and when used in the right population, virtual care may enhance communication, reduce PPE use, and reduce exposure risk. However, during periods where diagnostic evaluation, direct communication, or empathy are paramount, in-person care remains preferable. The future of telemedicine must be individualized and patient centered.

### Conclusions

In summary, our experience suggests that intrahospital telemedicine is feasible and well received. It can be used by a select subpopulation and effectively reduces infectious exposure and PPE usage, and can support communication, though with the caveat of a more limited capability for empathetic support and need to improve usability in a patient-centered manner.

## Appendix

Multimedia Appendix 1 Supplemental data.

## Abbreviations

EHR: electronic health record
PPE: personal protective equipment
