# Supplementary material for: Evaluation of an Intrahospital Telemedicine Program for Patients Admitted With COVID-19: Mixed Methods Study
Source: J Med Internet Res. 2021 Apr 29;23(4):e25987. doi: 10.2196/25987 (PMC8086788; doi:10.2196/25987)
Supplement: Multimedia Appendix 1 [file jmir_v23i4e25987_app1.docx]

**Multimedia Appendix 1.**

**Figure S1. Distribution of daily PPE use and documented prolonged contact by patient.**


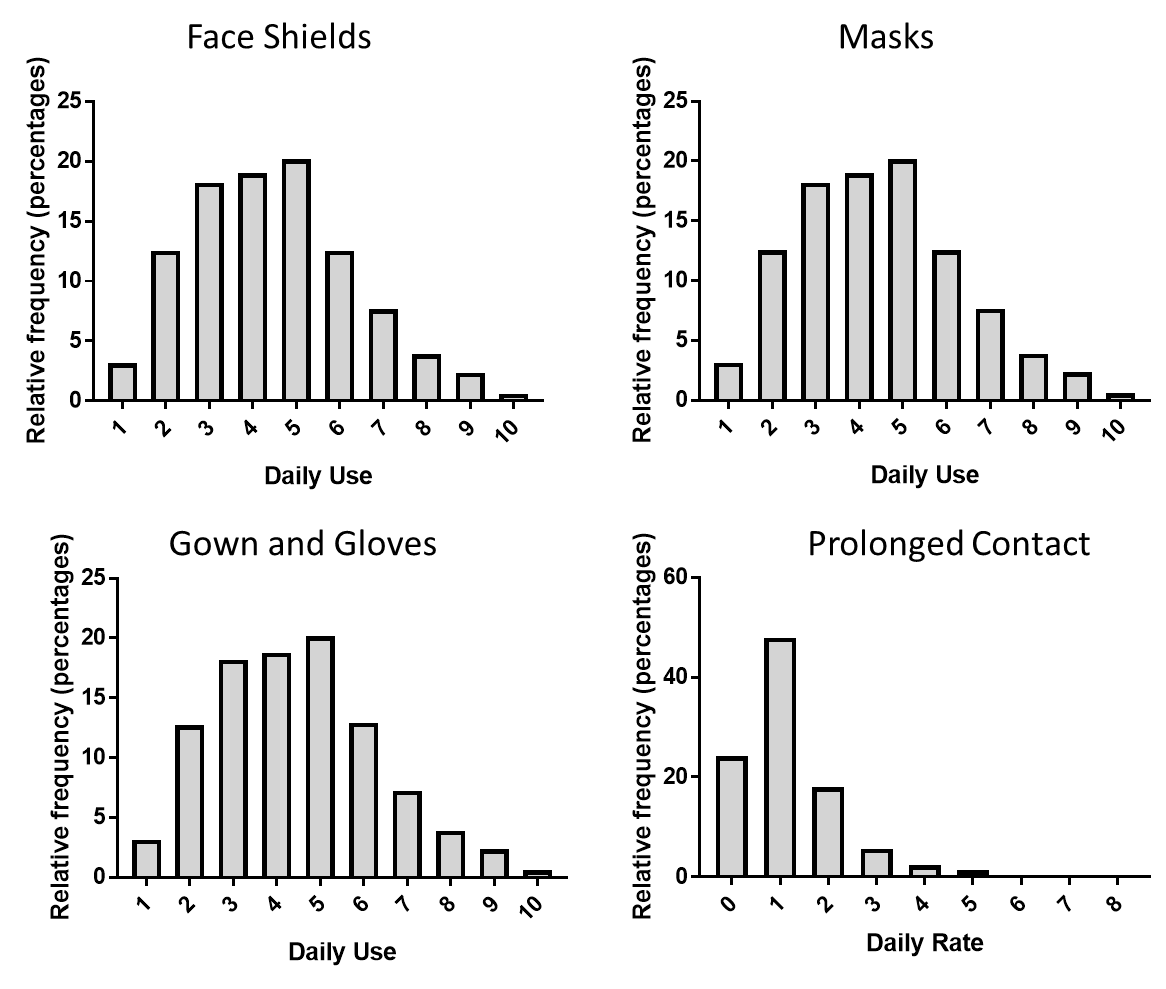


**Table S1.** Frequency of barriers experienced by providers who were unsuccessful at using virtual care.

|  | **Never** | **0-25% of the time** | **25-50% of the time** | **50-75% of the time** | **Always** |
| --- | --- | --- | --- | --- | --- |
| Patient acuity warrants active management | 0 (0.0%) | 3 (27.3%) | 5 (45.5%) | 3 (27.3%) | 0 (0.0%) |
| Language barrier | 1 (9.1%) | 2 (18.2%) | 1 (9.1%) | 6 (54.5%) | 1 (9.1%) |
| iPad is not turned on or the virtual meeting is closed | 1 (9.1%) | 1 (9.1%) | 2 (18.2%) | 3 (27.3%) | 4 (36.4%) |
| Patient has chronic conditions that prevent use | 1 (9.1%) | 5 (45.5%) | 2 (18.2%) | 3 (27.3%) | 0 (0.0%) |
| No device for either provider or patient | 2 (17.2%) | 4 (36.4%) | 2 (17.2%) | 1 (9.1%) | 2 (17.2%) |
| Physician preference | 3 (27.3%) | 4 (36.4%) | 3 (27.3%) | 0 (0.0%) | 1 (9.1%) |
| Patient preference | 4 (36.4%) | 5 (45.5%) | 1 (9.1%) | 0 (0.0%) | 0 (0.0%) |

**Table S2.** Comparison of survey responses of overall sentiment of inpatient.

| **Respondent** | **Question** | **Mean** | **SD** |
| --- | --- | --- | --- |
| **Patients** | I use Facetime or other video chat programs in my daily life | 2.13 | 1.5 |
|  | Virtual visits improved my ability to communicate with my care team | 2.73 | 0.96 |
|  | I felt that use of virtual communication reduced the risk of exposing my medical team to COVID-19 | 3.6 | 0.51 |
|  | Being able to use virtual communication allowed me to feel less isolated | 2.93 | 0.8 |
|  | I think continuing to use virtual visits in the hospital is a good idea | 3.13 | 0.52 |
|  |  |  |  |
| **Clinicians** | I had personally used telemedicine with a patient prior to this initiative | 1.65 | 1.44 |
|  | Using the ipad/remote assessment improves my ability to communicate with patients under isolation | 2.69 | 0.97 |
|  | Using the ipad/remote assessment reduced my exposure risk and use of PPE | 3.19 | 0.85 |
|  | iPad/Remote assessment should be expanded to other patients with COVID-19 | 2.84 | 0.96 |
|  |  |  |  |
| **Clinicians with use failure** | I have had previous experience with telehealth technology | 1.58 | 1.16 |
|  | In ideal state, virtual care/inpatient telehealth can limit risk of COVID exposure while providing effective care | 3.42 | 0.9 |
|  | In ideal state, virtual care/inpatient telehealth can improve family involvement in patient care | 3.41 | 1 |
|  | Virtual care/inpatient telehealth has the potential to improve care delivery | 3.25 | 0.62 |
